# Towards actinide heterostructure synthesis and science

**DOI:** 10.1038/s41467-022-29817-0

**Published:** 2022-04-25

**Authors:** Cody A. Dennett, Narayan Poudel, Paul J. Simmonds, Ashutosh Tiwari, David H. Hurley, Krzysztof Gofryk

**Affiliations:** 1 Condensed Matter and Materials Physics, Idaho National Laboratory, Idaho Falls, ID 83415 USA; 2 Department of Physics, Boise State University, Boise, ID 83725 USA; 3 Micron School of Materials Science and Engineering, Boise State University, Boise, ID 83725 USA; 4 Department of Materials Science and Engineering, University of Utah, Salt Lake City, UT 84112 USA

**Keywords:** Single photons and quantum effects, Surfaces, interfaces and thin films, Materials science, Electronic properties and materials

## Abstract

Controlling dimensionality and strain in actinide heterostructures will provide unrivaled opportunities for exploring novel quantum phenomena. We discuss the promises, challenges, and synthesis routes for these actinide-bearing heterostructures with complex electron correlations for functional and energy materials.

Advances in the synthesis and integration of dissimilar thin-film materials in the form of heterostructures have enabled the practical exploration of emergent quantum behavior for computing, communication, and sensing applications. Controlling dimensionality and local strain in nanostructure heterojunctions has provided unprecedented opportunities for understanding and exploring quantum degrees of freedom and their potential for new electronic and optoelectronic technologies. To date, actinide-based materials have yet to be synthesized with the atomic precision necessary for quantum heterostructure integration, despite their promising strong electronic correlations and spin-orbit interactions.

## Heterostructure science

In his 2000 Nobel Lecture on semiconductor heterostructures, Herbert Kroemer observed that “the interface is the device”^1^. One creates a heterostructure by monolithically layering two or more materials, usually with the specific goal of taking advantage of the unique physical characteristics of the interfaces, or heterojunctions, between them. Much of the early work by Kroemer, Alferov, Woodall, and others in the 1960s focused on III–V semiconductors, specifically the closely lattice-matched GaAs/AlGaAs system. By varying the composition of neighboring layers, researchers created semiconductor heterojunctions with novel band alignments for controlling the transport of electrons and holes with unprecedented precision. The ability to manipulate the motion of these carriers resulted in a rapid proliferation of electronic and photonic devices. By reducing the separation between two heterojunctions to close to the de Broglie wavelength, scientists began to exploit quantum mechanical effects. Quantum wells a few nanometers wide confine electrons and dramatically modify their allowed energy states. Devices taking advantage of quantum confinement include the high electron mobility transistor with low noise and high gain and magnetic field sensors which exploit giant magnetoresistance.

**Fig. 1:**
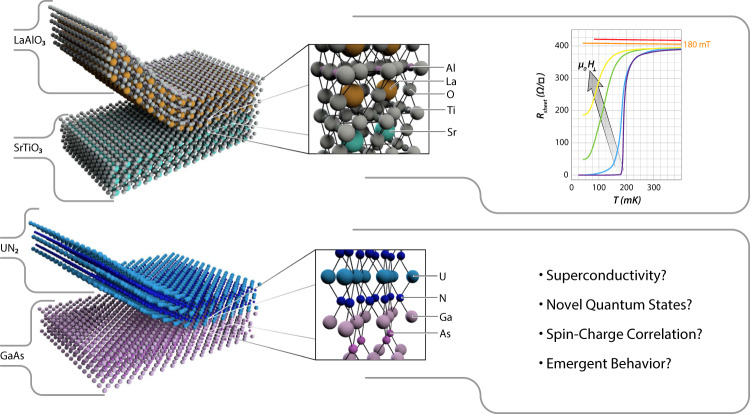
Novel physics have been observed in correlated heterostructures; the properties that will emerge from actinide heterostructures have yet to be discovered. Top: the SrTiO_3_/LaAlO_3_ heterojunction shows remarkable behavior, including the formation of a high-mobility, superconducting 2D electron gas at the interface of two band insulators. The right-most panel, adapted from Reyren et al., shows this superconducting transition and the critical field^28^. Bottom: potential actinide/semiconductor heterostructure interface between GaAs/UN_2_. With a lattice mismatch of ~0.7%, GaAs is a promising commercial substrate for actinide MBE synthesis, although appropriate temperature ranges for growth remain to be determined and a variety of substrates will need to be tested. What physics are exhibited by this and other actinide heterostructures can only be explored once a pathway for synthesis is established. The figure from Reyren et al. *Science* **317**, 1196–1199 (2007) has been adapted with permission from AAAS and the authors.

But it is not just device designers that benefit. Many breakthroughs in condensed matter physics are directly tied to thin-film heterostructures. The discovery of the integer quantum Hall effect^2^ was followed closely by that of the fractional quantum Hall effect^3,4^. These subtle quantum phenomena only became visible in heterostructures with exceptionally low electron scattering. Heterojunctions with constituents containing possible electron correlation, such as SrTiO_3_, have shown a variety of novel phenomena including a high-mobility electron gas at the interface of two insulating oxides, see Fig. 1^5^. More recent discoveries, from two-dimensional materials to topological insulators, are similarly reliant on our ability to produce high-quality thin films, combine them into heterostructures, and investigate the resulting properties of their surfaces and interfaces.

Returning to Kroemer’s quote, if the interface is king, then clearly interfacial quality is of paramount importance. Heterostructure devices may not operate as intended if their interfaces are not abrupt or clean. Decoherence of an electron’s fragile quantum states can occur if the confining heterojunctions are rough, or nonuniform in their atomic arrangement. The ability to create heterostructures with atomic precision is therefore essential. Simply sandwiching several materials together will not do. Techniques including molecular beam epitaxy (MBE) and chemical vapor deposition (CVD) enable users to synthesize high-quality heterostructures from a broad range of materials. Although these synthesis techniques may differ in terms of cost, growth rate, ease of use, etc., all permit the synthesis of single-crystal heterostructures for the discovery of new physics and the invention of novel devices.

## Expanding the elemental toolkit to actinides

The elemental toolkit for scientists exploring heterostructures is broad, comprising the majority of the periodic table with solutions for compounds including oxides, nitrides, borides, sulfides, and many more. However, a notable gap in this toolkit is the availability of actinide elements including thorium, uranium, and transuranics, despite their promise in exhibiting novel, emergent behavior. The lack of demonstrated technology in these systems stems primarily from constraints and controls with handling target-sized material volumes due to inherent radioactivity and material proliferation concerns. However, manageable solutions for the handling of radioactive target materials exist within appropriate facilities at certain universities and a wider selection of national research and development centers. Expanding the range of elements available for heterostructure synthesis and science to include the actinides will provide access to the unique physics moderated by correlated 5*f* electrons^6^.

While the electronic and structural properties of a large portion of the elements in the periodic table are well understood, this is not the case for actinide-bearing materials^7^. This is due to (1) strong electronic correlations (Columbic and Heisenberg exchange interactions), and (2) issues related to the hybridization and the number of 5*f* electrons in valence states that often exhibit duality through localized and itinerant behaviors^8^. These dual-nature electrons exhibit a complex interplay between multiple and competing interactions such as structural, orbital, charge, and spin degrees of freedom: the 5*f*-electron challenge. As a result, exotic electronic, optical, thermal, and functional properties are expected for actinide-based materials^9,10^. However, the tuning of these properties in the bulk is challenging and often introduces disorder through alloying or doping. By incorporating actinide materials into hetero- and nanostructures, new pathways for the control and design of quantum devices based on these unique 5*f* physics become possible.

The ability to synthesize epitaxial thin films and heterostructures from a wide range of quantum materials, including topological insulators and strongly correlated oxides, is now a rapidly expanding research area^11^. The exploration of actinide heterostructures would represent a novel direction for these efforts. By combining the potency and capabilities of interfacial engineering with the collective and emergent properties of quantum materials, quantum-matter heterostructures provide access to a new research space for condensed matter physics. In understanding, controlling, and exploiting the electronic, magnetic, and structural interactions of quantum heterostructures, a platform emerges not only for the exploration of novel quantum phenomena^12,13^, but also for the development of next-generation information, energy, and computing technologies. That promise is even more appealing in actinide systems, where 5*f*-electron orbitals are available for tuning and manipulation through perturbations of strain and local chemistry. For example, recent work in bulk actinide nitrides supports the idea that strain engineering of actinide-bearing topological insulators, in particular, is a promising avenue for exploration^14,15^. Expanding epitaxial synthesis capabilities to include 5*f*-bearing elements will create previously unobserved atomic and electronic arrangements, resulting in unique quantum states and new phases that cannot exist in the bulk.

## Actinide thin-film synthesis

This eventual goal of actinide heterostructure synthesis must target methodologies such as MBE or CVD that enable high-quality single-crystal growth and precise interface control. However, the work that has been conducted to date in actinide thin films suggests a staged approach incorporating other synthesis techniques will be necessary for achieving consistent and reliable heterostructures. In recent years, promising examples have emerged, primarily but not exclusively in uranium-bearing nuclear materials. A group currently operating at the University of Bristol employed reactive magnetron sputtering to produce epitaxial films of UO_2_ and nanocrystalline films of UO_2_, UN, U_2_N_3_. They studied a range of topics from antiferromagnetism^16^, to corrosion^17^, to phonon linewidths in irradiated specimens^18^. Researchers at Los Alamos National Laboratory have utilized two distinct methods, a polymer-assisted deposition technique to target a range of actinide oxides, carbides, and nitrides^19^ and, more recently, pulsed laser deposition to target epitaxial synthesis of uranium oxides: UO_2_, U_3_O_8_, and UO_3_^20^. Thin, sputtered layers of plutonium and its compounds have also been synthesized to better understand 5*f* electron localization in actinide thin films^21^. Other demonstrated actinide thin-film syntheses include physical vapor deposition of ThO_2_ on Ir ribbons^22^, DC magnetron sputtering of UO_2_ on Si^23^, DC sputtering of U in an N_2_ atmosphere to generate UN and U_2_N_3_^24^, and DC diode sputtering of U metal onto metallic substrates^25^. Such syntheses have accomplished important initial goals of verifying substrate compatibility and epitaxial lattice matching conditions, and have in turn revealed some of the fundamental optoelectronic properties of these materials. However, researchers have yet to demonstrate capabilities for targeted heterostructure growth and monolithic device integration based on actinide compounds.

To make the leap from isolated films to heterostructure engineering, a dedicated facility for actinide MBE is being established at the Idaho National Laboratory. From our knowledge of actinide vapor pressures, one can evaporate all elements of interest at MBE-compatible rates using either standard high-temperature effusion cells or electron-beam sources that are widely used for refractory metals. This synthesis capability will enable the precise control necessary for the generation of quantum materials through the exploration of dimensionality, chemistry, and strain in single crystal, epitaxial actinide films. The ability to control interactions between these variables will be fundamentally important to the discovery of novel emergent states and the eventual application of actinide-based or actinide-doped structures at the device scale. Near-term synthesis goals are being targeted in the nitride family, UN_*x*_ and ThN_*x*_. The future possibility of adding a second MBE chamber would permit us to expand into the growth of actinide oxides. To place any radiation or material protection concerns with respect to actinide functional devices in context, consider a standard americium-241 smoke detector. Reaching the level of radioactivity contained in this consumer product would require over one hundred 50 mm (2”) diameter, 0.5 mm thick UN wafers. This amount of material is orders of magnitude greater than what is used in typical device structures that are less than a micron thick. Under these conditions, the adoption of actinide-bearing quantum devices should not be hindered by their constituent materials.

## Coupled characterization and modeling

Once synthesized, it is likely that a full understanding of the complex properties and performance of actinide-bearing films and heterostructures will only be achievable through the use of an appropriate combination of characterization and modeling tools. Heterostructures in general can introduce extreme complexity for first principles models due to reduced symmetry and the strain-induced defects commonly generated in systems that are not perfectly lattice matched. While standard density functional theory (DFT) can accurately describe coherent, non-*f*-electron-bearing heterostructures, in the spirit of this Comment, such treatments would only be appropriate for thorium-bearing compounds. To first order, DFT + *U* is a good starting point to efficiently explore the phase space of actinide-bearing heterostructures. The DFT + *U* approach has been used successfully to treat local, correlated electron effects in 5*f-*^19^ and 4*f-*bearing^26^ bulk materials and epitaxial films. Although often used semiempirically, the DFT + *U* approach can be validated through comprehensive structural, electronic, phononic, and transport characterization. However, all implementations of DFT break down for strongly correlated electron systems, requiring computationally expensive solutions beyond DFT. An example of a next-order computational approach of current interest involves combining dynamical mean-field theory (DMFT) and DFT, yielding the so-called DFT + DMFT formalism. Additional advanced and in situ characterization methods such as reflection high-energy electron diffraction, scanning tunneling microscopy, scanning probe techniques, photoemission spectroscopy, magneto-transport, and more, could all lend detailed insight into both growth kinetics and evolving electronic and topological properties should they become available with actinide-compatible MBE synthesis^27^.

## Future possibilities in technology and physics

In the last half-century, the access to reliable, chemically-precise heterostructures has enabled volumes of novel physics to be documented and devices to be put into everyday practice. The epitaxial synthesis of single crystals on clean interfaces has allowed the extreme sensitivity of quantum materials to be controlled and engineered toward targeted applications. Extending this control, of chemistry, strain, local order, and more, to actinide materials with inherent complexity and correlation effects promises to drive fundamental breakthroughs in both technologies and physics. While MBE is a likely route to achieve these goals, challenges related to actinide material handling, substrate selection for lattice mismatch control and thermal stability, growth rates with low vapor pressure metals, and more, will surely be encountered. Nevertheless, the promise of observing and controlling these emergent phenomena is strong motivation to explore this path. Such capabilities will spur advancement in next-generation devices with applications in quantum information and science about which we can only speculate.
